# Exome Analysis Identifies a Novel Compound Heterozygous Alteration in *TGM1* Gene Leading to Lamellar Ichthyosis in a Child From Saudi Arabia: Case Presentation

**DOI:** 10.3389/fped.2019.00044

**Published:** 2019-02-21

**Authors:** Sami Raja Alallasi, Amal A. Kokandi, Babajan Banagnapali, Noor Ahmad Shaik, Bandar Ali Al-Shehri, Nuha Mohammad Alrayes, Jumana Yousuf Al-Aama, Musharraf Jelani

**Affiliations:** ^1^Department of Genetic Medicine, Faculty of Medicine, King Abdulaziz University, Jeddah, Saudi Arabia; ^2^Department of Dermatology, Faculty of Medicine, King Abdulaziz University, Jeddah, Saudi Arabia; ^3^Princess Al-Jawhara Albrahim Center of Excellence in Research of Hereditary Disorders, King Abdulaziz University, Jeddah, Saudi Arabia; ^4^Faculty of Applied Medical Sciences, King Abdulaziz University, Jeddah, Saudi Arabia; ^5^Centre for Omic Sciences, Islamia College Peshawar, Peshawar, Pakistan

**Keywords:** lamellar ichthyosis, exome sequencing, transglutaminase 1 gene, novel compound heterozygous, Saudi Arabia

## Abstract

**Background:** Lamellar ichthyosis is an autosomal recessive type of rare skin disorders characterized with defective epidermis leading hyperkeratosis with brownish-gray scales over the body. These patients are born as collodion babies and may also exhibit additional features like erythema, ectropion, and eclabium. This disease is mainly caused by homozygous and compound heterozygous alterations in transglutaminase 1 encoding gene (*TGM1*), which is located on 14q12.

**Case presentation:** This study reports the genetic analysis of a 4-year Saudi girl presenting lamellar ichthyosis. She was the first child of unrelated parents. The family had no previous history of the disease phenotype. She was born as a collodion baby without any prenatal complications. At the time of this study she had developed rough scaly skin on her legs, arms and trunk regions with thick palms and soles. Whole exome sequencing (WES) followed by Sanger sequence validation identified a novel compound heterozygous variant in *TGM1* gene. The paternal variant was a missense transition (c.1141G>A; p.Ala381Thr) present at exon 7, while maternal variant (c.758-1G>C) was present at the intron4-exon5 boundary. To the best of our knowledge these variants had not been reported before in *TGM1* gene.

**Conclusion:** In isolated and inbred populations, homozygous variants are identified more frequently; however, our results suggest that compound heterozygous variants should also be considered especially when the marriages are not consanguineous.

## Introduction

Lamellar ichthyosis (LI, MIM 242300) is one of the non-syndromic forms of ichthyoses inherited in autosomal recessive form. At birth, babies are covered with collodion-like membranes which sheds later and leaves scales all over the body (1). Severity of scaling differs from patient to patient. In general, patients have large thick scales which are either dark gray or brown in color. They may exhibit very milder form of erythema. However, palmoplantar keratoderma is more frequent while erythroderma is not consistent with LI (2). In some cases alopecia is also accompanied (3).

Genetic analyses of familial cases have reported eight genes responsible for LI phenotype. These genes include ATP binding cassette subfamily A member 12 (*ABCA12*, MIM 607800) at chromosome 2q35 (4), arachidonate lipoxygenase 3 (*ALOXE3*, MIM 607206) located at 17p13.1 (5), arachidonate 12-lipoxygenase (*ALOX12B*, MIM 603741) located at chromosome 17p13.1 (6), ceramide synthase 3 (*CERS3*, MIM 615276) located at 15q26.3 (7), cytochrome P450 family 4 subfamily F member 22 (*CYP4F22*, MIM 611495) located at chromosome 19p13.12 (8), NIPA like domain containing 4 (*NIPAL4*, MIM 609383) located at chromosome 5q33.3, patatin like phospholipase domain containing 1 (*PNPLA1*, MIM 612121) located at chromosome 6p21.31 (9) and transglutaminase 1 (*TGM1*, MIM 190195) located at chromosome 14q12 (10, 11).

Among the non-syndromic forms of autosomal recessive ichthyoses, *TGM1* alterations have been identified more frequently as compared to other known genes of LI (12). Previously, it was considered that mostly homozygous variants lead to non-availability of transglutaminase enzyme which in turn disrupts cornified cell envelope formation and thus result in skin barrier defect (3, 13). However, recently, a study from Japan has shown that compound heterozygous variants also lead to severe form of LI phenotype (14).

## Case Presentation

### Methods

In this study, we investigated a Saudi Arabian family for the disease variant of lamellar ichthyosis phenotype. We applied a combination of strategies covering whole exome sequencing (WES) to identify list of altered genes involved in autosomal recessive ichthyosis (Table 1) and then targeted Sanger sequencing (SS) of the short listed variants retrieved from WES analysis.

**Table 1:** List of candidate genes for autosomal recessive congenial ichthyosis.

**No**	**Gene Name**	**Ensemble reference ID**	**OMIM**	**Chromosome**
1	GJB2	ENSG00000165474	121011	Chr13
2	^α^ARCI9	Not known	615023	Not known
3	^α^ARCI7	Not known	615022	Not known
4	ABCA12	ENSG00000144452	607800	Chr2
5	ALOXE3	ENSG00000179148	607206	Chr17
6	CASP14	ENSG00000105141	605848	Chr19
7	TGM1	ENSG00000092295	190195	Chr14
8	GJA1	ENSG00000152661	121014	Chr6
9	ST14	ENSG00000149418	606797	Chr11
10	SDR9C7	ENSG00000170426	609769	Chr12
11	ALOX12B	ENSG00000179477	603741	Chr17
12	NIPAL4	ENSG00000172548	609383	Chr5
13	PNPLA1	ENSG00000180316	612121	Chr6
14	SULT2B1	ENSG00000088002	604125	Chr19
15	CERS3	ENSG00000154227	615276	Chr15
16	CYP4F22	ENSG00000171954	611495	Chr19
17	LIPN	ENSG00000204020	613924	Chr10
18	KRT10	ENSG00000186395	148080	Chr17
19	SLC27A4	ENSG00000167114	604194	Chr9
20	ELOVL4	ENSG00000118402	605512	Chr6
21	ANOS1	ENSG00000011201	300836	ChrX
22	LAMC1	ENSG00000135862	150290	Chr1
23	PEX7	ENSG00000112357	601757	Chr6
24	CYP4V2	ENSG00000145476	608614	Chr4
25	SGPL1	ENSG00000166224	603729	Chr10

α *NCBI has written this abbreviation based on the disease type. Gene is still known for these types*.

Genomic DNA of the index patient (II-1) was subjected to bidirectional WES analysis at 100x resolution with 150 base pairs reads length. Sureselect target enrichment system (Agilent Technologies, USA) was used to prepare 6 Mb WES library from 2 μg genomic DNA of the patient. The 100 bases long amplified libraries were then sequenced on NOVASEQ6000 (Illumina, USA) using Macrogen Inc. South Korea NGS sequencing facility. The raw data along with the variant call files were further analyzed for the causative gene/variant(s).

The wild type genomic sequence of TGM1 gene was obtained from Ensembl Genome Browser (https://www.ensembl.org/Homo_sapiens/Gene/Summary?g=ENSG00000092295). Selected variants were then Sanger sequenced using primers (Table 2) designed by Primers3plus software (www.bioinformatics.nl/primer3plus). The target exons were PCR amplified from genomic DNA samples with GoTaq^®;^ green mater mix (Promega, USA). The single stranded sequencing products were synthesized with BigDye terminator v3.1 cycle sequencing kit and run directly on ABI 3500 Genetic Analyzer (Life Technologies, USA). The sequences files were analyzed for mismatches using BioEdit editor version 7 software (www.mbio.ncsu.edu/BioEdit/bioedit.html).

**Table 2:** List of primers used for the novel *TGM1* variants' Sanger validation.

**No**	**Primer name**	**Sequence^α^**	**Ta**	**Product size (bg)**
1	TGM1_Ex5F	AGCCCAGGGTCACACAGCCA	63	636
2	TGM1_Ex5R	CGAGGCAGCAGGCACACACA	63	
3	TGM1_Ex5F2	GCTACAGCCAATCTCCTCCA	60	345
4	TGM1_Ex5R2	CCAGCTCCTCTGGGTGTATG	60	
5	TGM1_Ex7F	CCATCAGCGTGGGTGGGCAG	65	195
6	TGM1_Ex7R	AGCCACATCTGGGCAGGGCT	65	

α *The sequences are written from 5′ to 3′; Ta, annealing temperature; bp, base pairs*.

Variant effect predictor (VEP) toolset was used to predict the potential pathogenicity of the selected *TGM1* variants. The detailed description of VEP in terms of input, output and principle is described elsewhere (15). From the output results, we selected Mendelian Clinically Applicable Pathogenicity (M-CAP) score (for missense variant) and Max Ent Scan scores (for splice site variant) to understand the variant consequences following the standard variant annotation terms of “sequence ontology.” We further tried to understand the structural consequences of p.Ala381Pro variant using 3 dimensional TGM1 protein model. Owing to the lack of experimentally solved TGM1 structure, we took the reference 3D structure (PDB ID- 1GGT) of human blood coagulation factor xiii (with a resolution of 2.65 Å, 43.6% identity, Z-score of 23.44 and 710 aa length) to create the both native and mutant 3D molecular models of TGM1 protein (NP_000350) using Swiss modeler (https://swissmodel.expasy.org/). These molecular models were analyzed for structural differences in terms of hydrogen bonding characters, solvent accessibility (16), stability (17), and structural deviations (18).

## Results

### Clinical History

**Figure 1:**
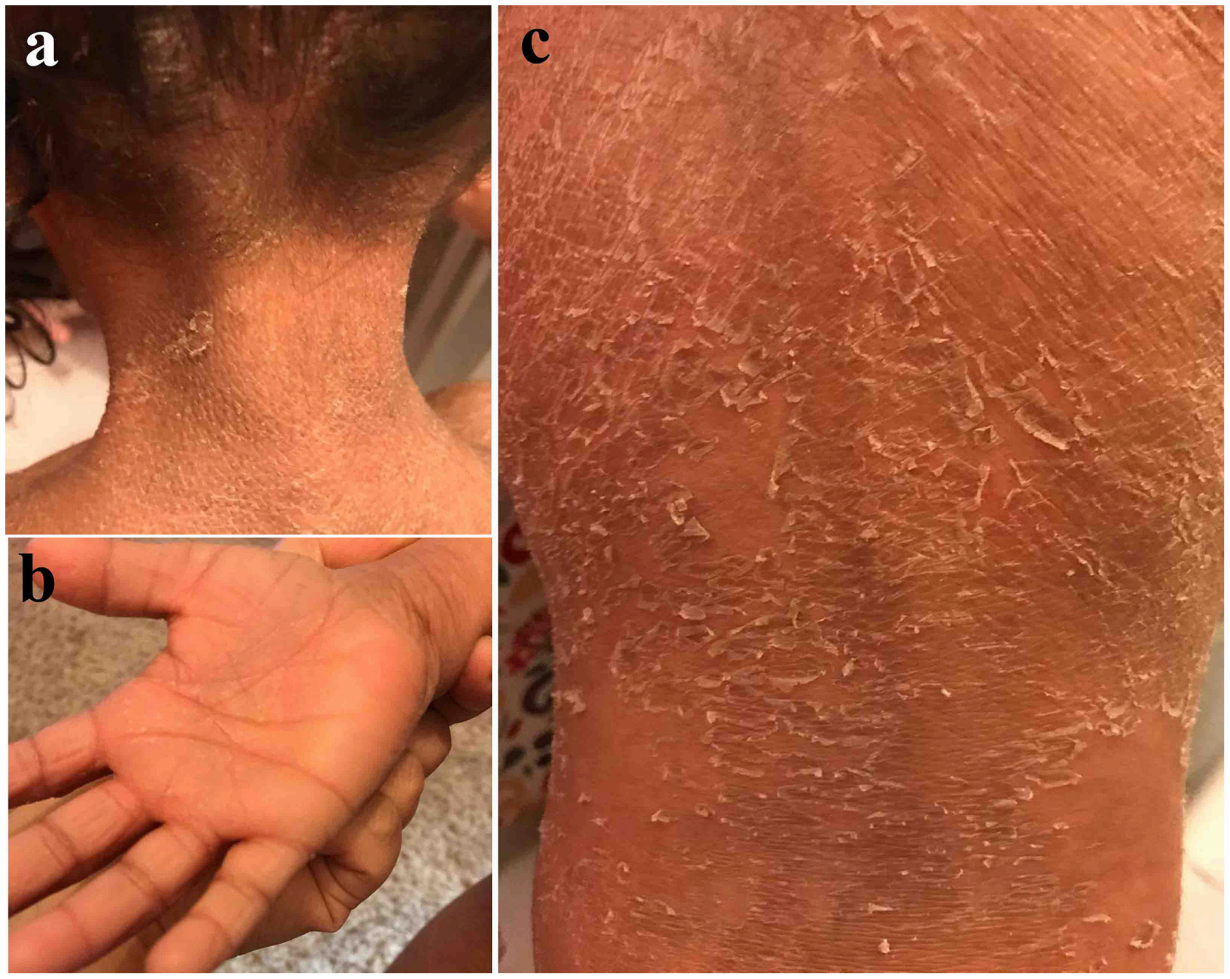
Clinical presentation of patient II-1 in family A. Note the rough and dry skin with over the scalp and neck **(a)** palmoplantar keratoderma and exfoliation, fissuring on the palms **(b)**. The upper back area is more prominent with large thick and brownish scales **(c)**.

The affected girl II-1 (4 years at the time of study), was born to a non-consanguineous union. The parents (I-1 and II-2) narrated that they had no record of similar patient in their families. She was born as collodion baby on term with a normal pregnancy. She had developed rough and dry skin with palmoplantar keratoderma (thickening of palms and soles). She had exfoliation, fissuring on the palms but hyper-linearity was not evident. Her upper back was more prominent with large thick and brownish scales (Figure 1). These scales were also present on arms and lower limbs. She had sparse hair on scalp. The primary dentition appeared normal. There was no associated anomaly of other body parts or functions.

### Exome Sequencing

There were 103,605 variants identified in the initially analyzed variant call files of WES data. Out of these, 171 variants were present in those genes which had previously been known for autosomal recessive congenital ichthyosis (Table 1). They included five in 3′ untranslated regions, one in 5′ untranslated region, two in downstream gene position, three in intergenic regions, 119 in introns, 16 in coding exons (missense), three at splicing regions, 20 synonymous and two upstream gene variants.

### Potential Candidates' Prioritization

Based on zygosity and minor alleles frequency, variants list was further analyzed. About 120 variants were in homozygous and 51 were in heterozygous conditions. Variants with minor allele frequencies equal or < 0.01 were 18 only (Table S1). Out of these 18, two variants (a splicing c.758-1G>C and a missense c.1141G>A; p. Ala381Thr of *TGM1* gene NM_000359.2) were selected for Sanger validation based on clinical resemblance of our patients to the previously reported LI patients with *TGM1* alterations. They were present at the splicing region exon 5 and in the coding region of exon 7 of *TGM1* gene. Both these variants were heterozygous, and the compound heterozygosity was assumed in the affected sibling. Sanger sequencing was performed to validate the affected alleles segregation.

### Sanger Sequencing of *TGM1* Variants

**Figure 2:**
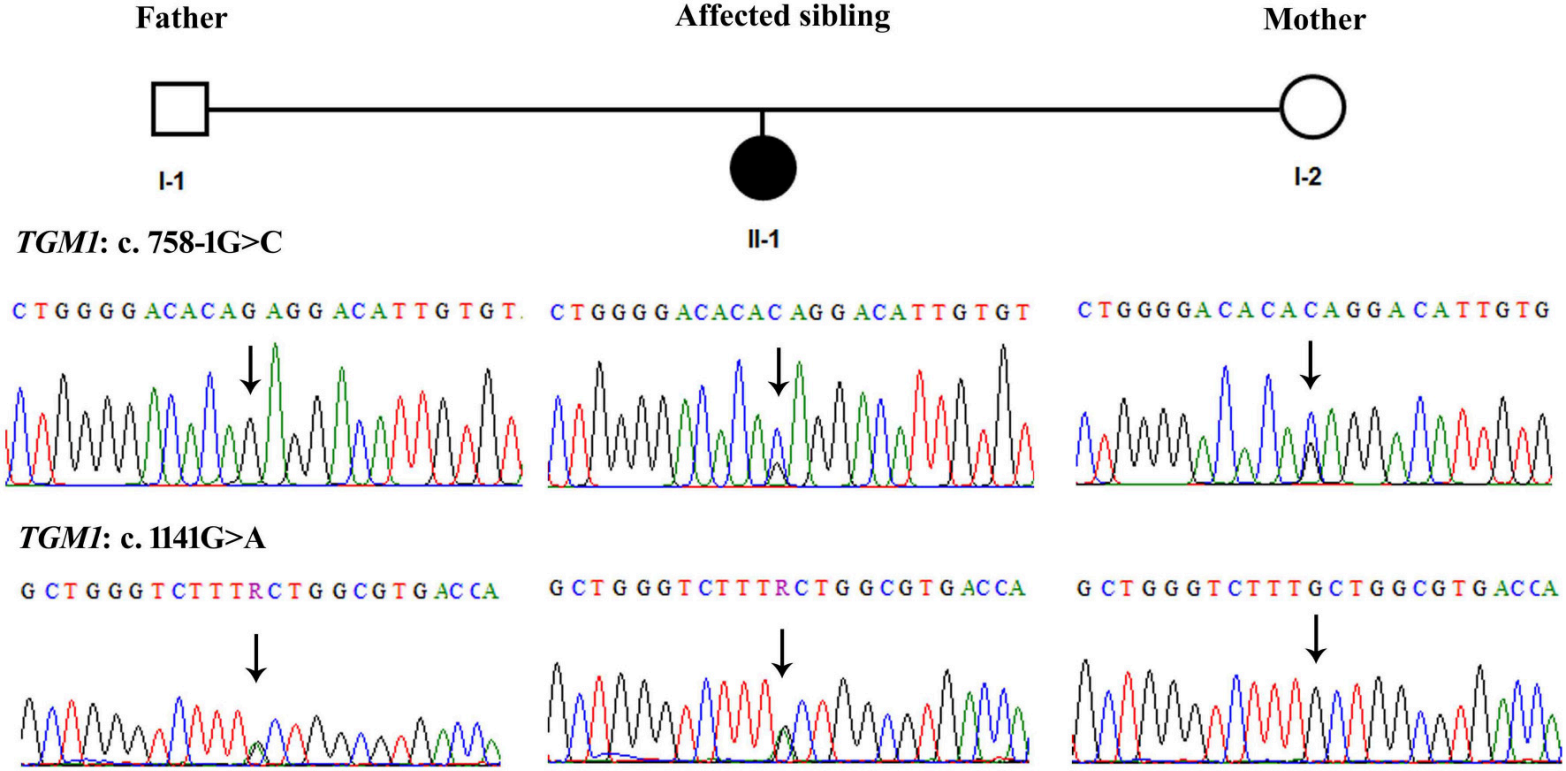
Sanger sequencing of the *TGM1* variants showing segregation of c.758-1G>C (Intron4-Exon5 boundary) from mother and c.1141G>A (Exon 7) segregation from father. The arrows indicate respective nucleotides in the sequences. Right panels show that father is wild type for c.758-1G>A and carrier for c.1141G>A. The right panels show that mother is carrier for c.758-1G>A and wild type for c.1141G>A. The middle panels show that affected sibling has both variants in heterozygous form.

Sanger sequencing confirmed that the missense variant c.1141G>A; p. Ala381Thr in exon 7 of *TGM1* gene segregated from the father (I-1) and alternatively the splice site variant at exon 5 was carried by the mother (I-2) (Figure 2). Thus, the index patient had compound heterozygous status for paternal variant in exon 7 and maternal variant in exon 5. To the best of our knowledge both these variants were novel and were not reported in published literature previously. They were also not listed 1,000 genome (www.internationalgenome.org/) nor in ExAc database (exac.broadinstitute.org/) and nor greater middle eastern genome database (igm.ucsd.edu/gme/). These variants were also not found in >200 chromosomes of the ethnically matched healthy volunteers.

### Computational Analysis of *TGM1* Variants

**Figure 3:**
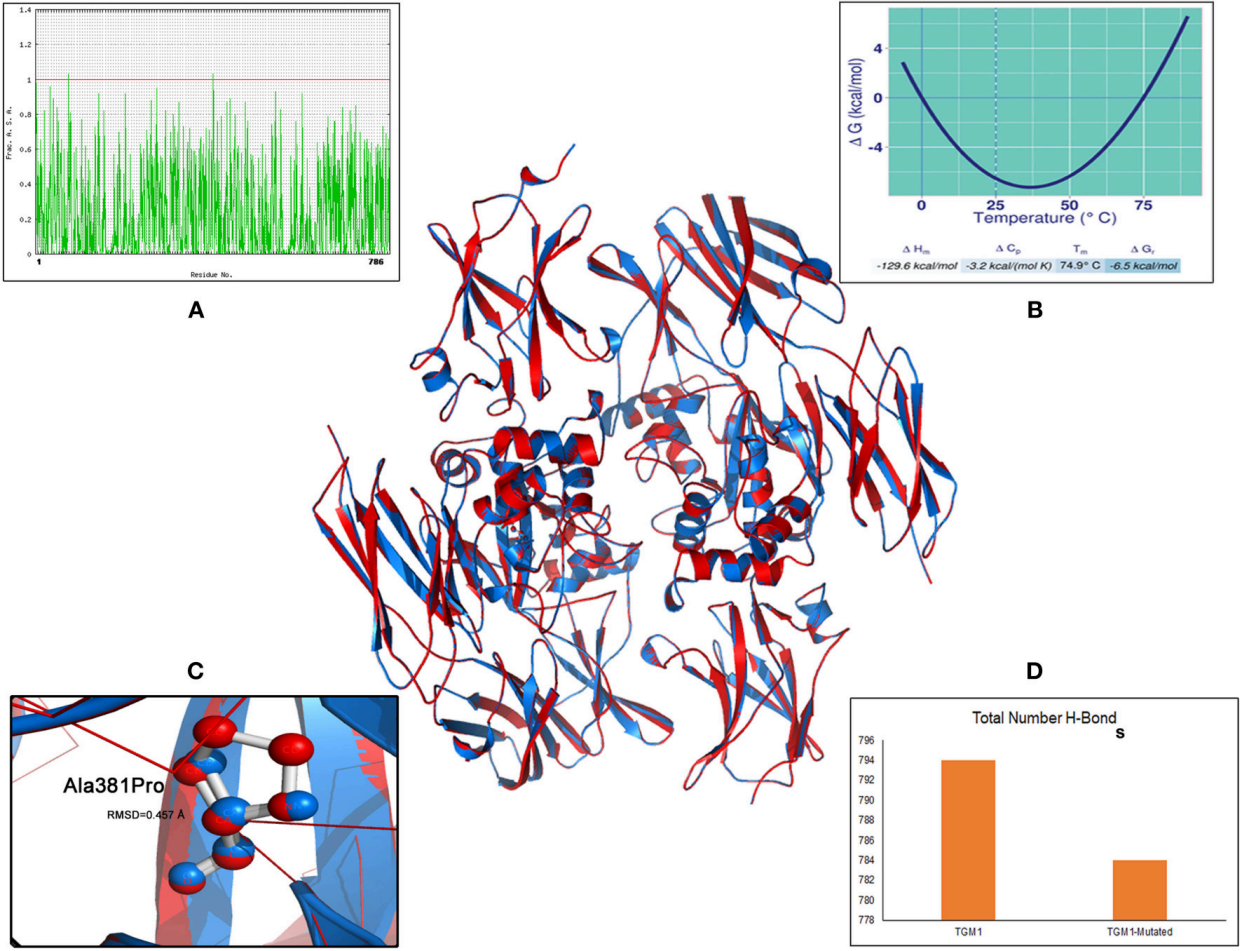
Molecular view of TGM1 altered (p.Ala381Pro) protein model and its biophysical characteristics. Solvent accessibility graph **(A)** Stability curve **(B)** RMSD value **(C)** number of hydrogen bonds **(D)**.

The MaxEntScan prediction of c.758-1G>C shows that the wild type “G” and mutant “C” nucleotides possess entropy scores of 2.565 and −5.499, respectively. The “C” nucleotide is likely to disturb the splice site junction by 80.46%, hence has potential to alter the normal splicing of *TGM1* transcript. Protein analysis of the missense variant (c.1141G>A; p.Ala381Pro) (Figure 3), showed an M-CAP score of 0.736, and any variant with an M-CAP score of >0.50 is considered highly deleterious to the normal protein structure and function (19). Thus, p.Ala381Pro variant is most likely pathogenic in our patient.

The specific impact of p.Ala381Pro on TGM1 protein can be better understood by examining additional structure-based features like root mean square deviation (RMSD), stability, solvent accessibility, and hydrogen bonding. The 3-dimensional protein model analysis has suggested that the mutant Proline residue brings a RMSD value of 0.45 Å value of 381th position in the TGM1 protein. RMSD refers to the quantitative metric of structural resemblance between native and altered protein molecules. The substituted proline residue seems to bring a destabilizing effect on TGM1 protein, by shifting the Gibbs free energy values (ΔΔG) toward negative range (−6.53 Kcal/mol). The solvent accessibility value (SAV) of mutant Proline residue in TGM1 is abolished due to its buried form compared the native Alanine residue (SAV is 1.83%), which exists in exposed state. Hydrogen (H) bonding analysis revealed that the number of H-bonds are reduced in mutant state (Proline = 783 bonds) compared to its native form (Alanine = 793 bonds) (Figure 3).

## Discussion

Exome sequencing analysis has widely been used as an efficient molecular diagnostic tool in single gene disorders. Marriages within closer relatives are always at high risk for autosomal recessive disorders. However, the situation becomes more worrying for those parents who are not cousins and they have first child with a life threatening autosomal recessive disorder, lamellar ichthyosis.

Three genes including *TGM1* have been reported previously for LI patients from Saudi Arabia and neighboring Arab countries. First genetic analysis of Saudi patients was carried out through homozygosity mapping followed by targeted *TGM1* gene's Sanger sequencing (20). More efficient approach of whole exome sequencing was carried out in United Arab Emirates families, in which three previously reported *TGM1* variants were identified. The authors concluded that *TGM1* was one of the most frequent cause of LI in the Middle East (20, 21). Another extensive study from Scandinavian countries has also found *TGM1* alterations in most of the LI patients (22).

The transglutaminase-1 (TGM-1) enzyme is found in the epidermis, where it is responsible for cross linking the keratin precursors and to form the cornified cell envelope. This cross linking provides mechanical strength and stability to the epidermal layer of skin. The 817-aa long TGM-1 is composed of 4 functional domains including Transglut N (117–235 aa), Transglutaminase/protease-like homologs domain (TGc) (348–468 aa), Transglut C domain (587–691 aa & 699–796 aa). TGM1 protein deficiencies also have a role in the compensatory overexpression of other transcripts involved in skin barrier repair. This upregulation of autosomal recessive congenital ichthyosis genes might reflect a compensatory induction of omega-O-acyl ceramides which are part of a global barrier repair response in the patient's epidermis (23).

The p.Ala381Pro variant identified in our case lies in the main catalytic (TGc) domain of TGM1 protein. This might affect several structural disturbances by altering conformation, stability, solvent accessibility and hydrogen bonding properties of this protein. All these biophysical parameters play a very important for the maintenance of polypeptide folding and 3 dimensional conformation of protein structure, in addition to determining its metal binding characteristics (18, 24, 25). These structural disturbances might induce altered Ca^2+^ binding properties and misfolding of the polypeptide in the endoplasmic reticulum (ER). Which would eventually lead to ubiquitinoylation and release of TGM1 from ER instead of being trafficked to cell membrane. The abnormal ubiquitinoylation and accumulation in perinuclear structures forming aggresomes, could ultimately lead to the lower levels of transglutaminase activity in the cell membranes (26).

The c.758-1G>C splice site variation could abrogate the normal AG acceptor site at exon 5-intron 5 junction, resulting in the production of extra-long m-RNA. There are increasing evidences which have showed that point variants in 3′ donor splice site, in particular the variants involving G residue at −1 are rather rare (27). The acceptor splice variant reduces the pairing of acceptor splice site with complimentary site in U1snRNP complex, which is the first step in mRNA splicing (28). Therefore, variants acceptor splice sites might lead to intron-4 retention or exon-5 skipping or cryptic site activations as described elsewhere (29).

## Conclusion

Based on our molecular genetic findings, we expect that the lamellar ichthyosis patient we studied here, has inherited two defective alleles (missense and splice site variants) from her parents. Both splice site and missense variants might have contributed to loss-of-function of transglutaminase 1 enzyme in the patient. The defective or reduced availability of TGM1 enzyme may result in the formation of an abnormally functioning stratum corneum, the outermost epidermal layer with defective intercellular lipid layers (30), thus leading to skin abnormalities and clinical manifestations of LI.

## Study Limitations

This study sincerely admits that lack of functional biology assays is one of the main limitations to ascertain the causative role of *TGM1* gene in this family. However, given the location of p.Ala381Pro in TGc domain, whose main role is in enzyme catalysis, we expect it to be the main causative factor for lamellar ichthyosis in this family. *TGM1* molecular data produced in this study might have important implications for the prenatal diagnosis, disease prevention in risk group individuals and pave a way for future therapies aimed at personalized medicine.

## Ethics Statement

Prior to start of this study an approved consent was signed by the family members. All the participants voluntarily provided their genomic DNA samples and the study was approved according to Helsinki's Declaration by ethical committee of medical and research, Faculty of Medicine, King Abdulaziz University Jeddah under the project reference number 24-14.

## Author Contributions

SA performed laboratory experiments and compiled results. NS and BB performed 3D protein modeling analyses and wrote the results and discussion. AK wrote and provided clinical history of the enrolled patients. NA and BA-S collected samples and performed Sanger sequencing data analysis. JA-A critically reviewed the manuscript. MJ designed the study, analyzed WES data, wrote, and finalized the manuscript.

### Conflict of Interest Statement

The authors declare that the research was conducted in the absence of any commercial or financial relationships that could be construed as a potential conflict of interest.
